# Percussion Entropy Analysis of Synchronized ECG and PPG Signals as a Prognostic Indicator for Future Peripheral Neuropathy in Type 2 Diabetic Subjects

**DOI:** 10.3390/diagnostics10010032

**Published:** 2020-01-09

**Authors:** Hai-Cheng Wei, Na Ta, Wen-Rui Hu, Sheng-Ying Wang, Ming-Xia Xiao, Xiao-Jing Tang, Jian-Jung Chen, Hsien-Tsai Wu

**Affiliations:** 1 School of Electrical and Information Engineering, North Minzu University, No. 204 North Wenchang Street, Yinchuan 750021, Ningxia, China; wei_hc@nun.edu.cn (H.-C.W.); ta_na@nmu.edu.cn (N.T.); 20187144@stu.nun.edu.cn (W.-R.H.); 20187150@stu.nun.edu.cn (S.-Y.W.); xiao_mx@nmu.edu.cn (M.-X.X.); 2 Basic Experimental Teaching and Engineering Training Center, North Minzu University, No. 204 North Wenchang Street, Yinchuan 750021, Ningxia, China; 3 School of Science, Ningxia Medical University, No. 1160 Shengli Street, Yinchuan 750004, Ningxia, China; tangxj@nxmu.edu.cn; 4 Taichung Tzuchi Hospital, The Buddhist Tzuchi Medical Foundation, Taichung 42743, Taiwan; cjjwei@tzuchi.com.tw; 5 Department of Electrical Engineering, Dong Hwa University, No. 1, Sec. 2, Da Hsueh Rd., Shoufeng, Hualien 97401, Taiwan

**Keywords:** type 2 diabetes, diabetic peripheral neuropathy (DPN), electrocardiogram (ECG), photoplethysmography (PPG), percussion entropy index (PEI)

## Abstract

Diabetic peripheral neuropathy (DPN) is one of the most common chronic complications of diabetes. It has become an essential public health crisis, especially for care in the home. Synchronized electrocardiogram (ECG) and photoplethysmography (PPG) signals were obtained from healthy non-diabetic (*n* = 37) and diabetic (*n* = 85) subjects without peripheral neuropathy, recruited from the diabetic outpatient clinic. The conventional parameters, including low-/high-frequency power ratio (LHR), small-scale multiscale entropy index (MEI_SS_), large-scale multiscale entropy index (MEI_LS_), electrocardiogram-based pulse wave velocity (PWV_mean_), and percussion entropy index (PEI), were computed as baseline and were then followed for six years after the initial PEI measurement. Three new diabetic subgroups with different PEI values were identified for the goodness-of-fit test and Cox proportional Hazards model for relative risks analysis. Finally, Cox regression analysis showed that the PEI value was significantly and independently associated with the risk of developing DPN after adjustment for some traditional risk factors for diabetes (relative risks = 4.77, 95% confidence interval = 1.87 to 6.31, *p* = 0.015). These findings suggest that the PEI is an important risk parameter for new-onset DPN as a result of a chronic complication of diabetes and, thus, a smaller PEI value can provide valid information that may help identify type 2 diabetic patients at a greater risk of future DPN.

## 1. Introduction

The prevention and early detection of diabetes mellitus (DM) in high-risk subjects such as those with metabolic syndrome have become important issues in preventive medicine and public health [1,2]. As for type 2 diabetic patients, the outcomes of microvascular and macrovascular complications can be grievous. Diabetic peripheral neuropathy (DPN) is not only one of the most common complications of chronic diabetes, but also a leading cause for disability due to foot amputation and ulceration, fall-related injury, and gait disturbance. [3,4]. Not only is the quality of life much lower among DPN patients but the mortality rate has also been shown to be higher than for DM alone [5]. To avoid developing a severe condition, blood glucose control is the most important issue for type 2 diabetic patients [6,7,8]. However, this is not easy to achieve, which is the reason many researchers have tried to develop some non-invasive methods and instruments [9] for type 2 diabetic patients to avoid the increased risk of developing atherosclerosis and autonomic nervous dysfunction [10,11,12,13].

Heart rate variability (HRV) using electrocardiography (ECG) is an assessment method of autonomic function and baroreflex sensitivity (BRS) [14]. The low-/high-frequency power ratio (LHR) is the autonomic function index for frequency domain analysis and it is considered to balance the reflection between sympathetic and parasympathetic activity changes [14,15,16]. However, the conventionally used frequency domain parameter is not always adequate for this purpose because of non-stationarity and the nonlinear physiological signals adopted [17,18,19]. Therefore, several new parameters based on nonlinear dynamics theory were recently applied to HRV studies on autonomic function and BRS [20,21,22]. Among these parameters, a small-scale multiscale entropy index (MEI_SS_) was adopted to evaluate the autonomic nervous activities and BRS based on a nonlinear approach, using only the R–R interval (RRI) datasets [20]. In recent years, a new percussion entropy index (PEI) using synchronized ECG and photoplethysmography (PPG) signals was used to assess the BRS complexity in healthy elderly and diabetic subjects related to autonomic function changes [21,22]. Moreover, in a recent study [23] on a modified PEI, the PPG signals were measured and the peak-to-peak interval (PPI) series were calculated at the fingertip as an indicator of DPN in the aged and diabetic patients. However, real-time processing was not possible for the PPI-based index presented in [23]. On the other hand, parameters including the large-scale multiscale entropy index (MEI_LS_) [20], in addition to pulse wave velocity (PWV_mean_) [24,25,26,27], were reported for atherosclerosis detection in type 2 diabetes mellitus.

From the viewpoint of data analysis, we propose that some of the above parameters (i.e., LHR, MEI_SS_, MEI_LS_, PWV_mean_, and PEI) could present highly significant differences between diabetic patients who underwent a synchronized ECG and PPG signals testing at baseline during the early stages of disease; these patients were then followed for a further six years, with and without DPN. Furthermore, this study was designed to apply synchronized ECG and PPG signals from a non-invasive instrument in predicting the development of peripheral neuropathy in type 2 diabetic patients. It is worth mentioning that PEI [21,22] has recently been introduced to assess the complexity of BRS, while the significance of smaller PEI values concerning the identification of subjects with type 2 diabetes who are more prone to developing diabetic neuropathy is unknown. This study aimed at testing the hypothesis that a smaller PEI value at the baseline measurement can provide valid information that may help identify type 2 diabetic patients at a greater risk of future DPN.

The rest of this paper is organized as follows. Section 2 describes the study population and the baseline examinations and protocol of measuring of synchronized ECG and PPG signals. A follow-up procedure and DPN status checking were addressed, followed by an explanation of the statistical analysis methods. As presented in Section 3, results from the five indices—LHR, MEI_SS_, MEI_LS_, PWV_mean_, and PEI—were first computed for diabetic subjects with peripheral neuropathy within six years (i.e., Group 3) for comparison with the healthy elderly subjects (i.e., Group 1) and diabetic patients without peripheral neuropathy (i.e., Group 2). Subsequently, three new diabetic subgroups (i.e., Groups A–C) with different periods of PEI values were identified for follow-up and Cox proportional Hazards model as well as goodness-of-fit test for relative risks analysis. Finally, a Cox regression analysis of risk factors for the incidence of DPN within six years of follow-up in diabetic patients was verified. In Section 4 and Section 5, the discussion of the results and conclusions from the present study are summarized, with suggestions for future work.

## 2. Materials and Methods

### 2.1. Study Design and Study Population 

#### 2.1.1. The Inclusion and Exclusion Criteria Were as Follows

Between July 2010 to March 2013, 128 subjects were enrolled for this study. All diabetic patients were recruited from the diabetes outpatient clinic of the Hualien Hospital (Hualien City, Taiwan), while healthy controls were recruited from a physical check-up program at the same hospital. All of the age-controlled healthy subjects had no personal or family history of cardiovascular diseases. Of the 128 volunteers, 6 were excluded due to a history of coronary heart disease, heart failure, ischemic stroke, peripheral arterial disease, chronic atrial fibrillation, or permanent pacemaker implantation. 

#### 2.1.2. Grouping

The remaining 122 subjects were then first divided into three groups, namely, healthy upper-middle-aged subjects (Group 1, age range: 40–79, number = 37), upper-middle-aged subjects diagnosed as having type 2 diabetes (Group 2, age range: 37–82, number = 58, glycated hemoglobin (HbA1c) ≥ 6.5%), and type 2 diabetic patients developing peripheral neuropathy within 6 years after baseline measurement (Group 3, age range: 44–77, number = 27) (Table 1). The baseline characteristics of these study subjects are presented in Table 1. Type 2 diabetes was diagnosed by either a fasting blood glucose concentration ≥126 mg/dL or HbA1c ≥ 6.5% [28]. Subsequently, the PEI values for diabetic patients—85 in total—in Groups 2 and 3 were arbitrarily divided into three new subgroups for the prognostication of subjects with type 2 diabetes who are more prone to develop DPN. That is, the diabetic subgroups were created on the basis of quartiles in the diabetic population distribution of the PEI—the upper 25% (i.e., Group A), the middle 50% (i.e., Group B), and the lower 25% (i.e., Group C).

#### 2.1.3. Ethical Issues, IRB, and Consent Form

The study was approved by the Institutional Review Board (IRB) of Hualien Hospital (Hualien City, Taiwan) [26,27] and Ningxia Medical University (Yinchuan City, Ningxia Province, China) Hospitals (No.2018-229). All subjects gave written informed consent. 

#### 2.1.4. Study Protocol

Each subject was required to refrain from caffeine-containing beverages and theophylline-containing medications for at least 8 h before the baseline data measurement. Before taking the measurements, all subjects were requested to sign informed consent forms and complete questionnaires on demographics and medical histories, as well as receive blood sampling for serum biochemical analysis.

A detailed explanation of the aim and procedures was provided, as well as the measurement of synchronized ECG and PPG signals to be used for follow-up study. The test subjects received a standardized medical examination by a doctor that consisted of anthropometric, physiological, and biochemical measures at baseline. The indices of atherosclerosis and autonomic nervous function were subsequently computed. All diabetic patients underwent regular clinic treatment and follow-up in an outpatient clinic for at least eight years (i.e., two years for DM identification and six years for the follow-up period). DPN was diagnosed as the presence of symptoms of numbness, tingling, or pain of distal extremities lasting for more than 3 months in the same diabetes outpatient department through neurophysiological study [29].

#### 2.1.5. Follow-up and DPN Status

The DPN status for the subjects in Group 3 at each follow-up stage was ascertained by questionnaire and clinical medical examinations. The screening DPN from type 2 diabetes patients at the baseline and follow-up periods was based on the presence of symptoms of numbness, tingling, or pain of distal extremities lasting for more than 3 months and a confirmed diagnosis by the clinic doctor (i.e., in accordance with neurophysiological study). The study population comprised a sample of 27 type 2 diabetes patients with DPN (aged 62.81 ± 1.71 years) who underwent a synchronized ECG and PPG signals measurement at baseline and then were followed for at least 6 years after the baseline measurement at the same hospital.

### 2.2. Baseline Measurements and Protocol of Measurement of Synchronized Electrocardiogram (ECG) and Photoplethysmography (PPG) Signals

**Figure 1:**
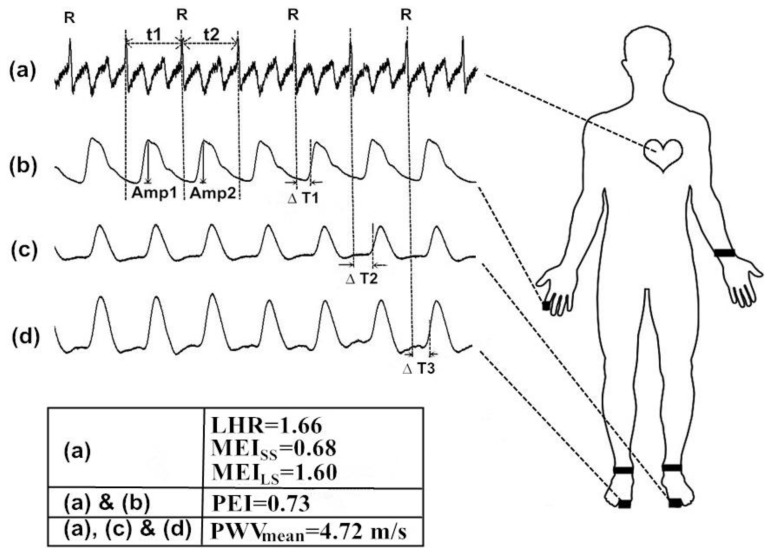
Schematic illustration of the measurements of six-channel electrocardiogram-based pulse wave velocity (ECG-PWV). The ECG and digital volume pulses (DVP) signals from one representative female subject in Group 1 with age of 44 and HbA1c of 5.1%. With (**a**) the R wave on Lead II of ECG, three parameters (i.e., low-/high- frequency power ratio (LHR), small-scale multiscale entropy index (MEI_SS_), and large-scale multiscale entropy index (MEI_LS_) were computed using only the RRI dataset. The synchronized ECGs (**a**) and the right index finger photoplethysmography (PPG) signals (**b**) were obtained for percussion entropy index (PEI) computation. With (**a**) the R wave on Lead II as a reference point, the time differences (ΔT_2_ (**c**) and ΔT_3_ (**d**)) for the second toe were obtained. The PWV_mean_ was calculated by dividing the distances from different points of reference (L) with ΔT (i.e., PWV = L/ΔT). The PWV_mean_, in the evaluation of the degree of atherosclerosis in the lower extremity of the body, was obtained by averaging the PWV values from both sides of the foot.

All measurements were performed over a period in the morning (i.e., 08:30–10:30). In addition, to minimize the potential errors in the infrared sensor readings arising from involuntary vibrations of the participants, all subjects were allowed to rest in a supine position for 30 min in a quiet room with a temperature maintained at 26 ± 1 °C. Blood pressure readings were obtained once over the left arm of the supine subjects using an automated oscillometric device (BP 3AG1; Microlife Corporation, Taipei, Taiwan) with an appropriate cuff size. A self-developed six-channel electrocardiography ECG-PWV-based system [26,27] was used to acquire 1000 successive recordings of the RRI signals and digital volume pulses (DVPs) within 30 min. To validate the application of the ECG-PWV system in assessing autonomic function, the RRI series was used for the LHR [14,16], MEI_LS_, and MEI_SS_ computations. Accordingly, the present study analyzed the RRI signals by dividing the MEI according to a small scale (MEI_SS_, mean value of sample entropy on a scale from 1 to 5) and large scale (MEI_LS_, mean value of sample entropy on a scale from 6 to 10) for comparison [20]. The DVPs of PPG with the R wave on RRI as a reference point could be used for the electrocardiogram-based pulse wave velocity (PWV_mean_) [26,27] and PEI [21,22] computations for assessing the autonomic function considering the degree of atherosclerotic change and autonomic function, respectively (Figure 1). In our previous study [21], changes in the BRS caused one to five cardiac cycle delays under the effects of fingertip DVP amplitude variations followed by synchronized RRIs. Accordingly, in obedience with the fluctuation tendency in the PEI computation, the percussion entropy had a length of the fluctuation pattern equal to two (i.e., PEI main contributor), and was expressed as BRS, while the percussion entropy, with a length of the fluctuation pattern equal to three (i.e., PEI major offset), was indicated as the biological complex system.

### 2.3. Statistical Analysis

The values are expressed as mean ± SD in Tables 1–3. The comparisons of the continuous valuables were analyzed using a Student’s unpaired *t* test with Bonferroni correction, and the differences between the categorical variables were assessed using a chi-square test. For the goodness-of-fit test and relative risk analysis, the PEI values were arbitrarily divided into three categories by the interquartile range method. The PEI was processed as both continuous and categorical variables and was undertaken in the Cox proportional hazards model to analyze the multivariate parameters according to the expansion of DPN. The relative risks (RR) were predicted with Cox regression analysis with corresponding 95% confidence intervals [30]. The following traditional risk factors for DPN were included as variables in the model: age, BMI, resting systolic and diastolic blood pressure, total cholesterol, triglyceride, waist circumference, pulse pressure, high-density lipoprotein cholesterol, low-density lipoprotein cholesterol, glycosylated hemoglobin, and fasting plasma glucose from baseline (i.e., PEI provided) to the end of the follow-up period (no longer than 6 years for each patient). According to the chi-square goodness-of-fit test in SPSS, the null hypothesis was rejected with a computed chi-square value larger than the level of significance. The Statistical Package for the Social Sciences (SPSS, version 14.0 for Windows, SPSS Inc., Chicago, IL, USA) was utilized for all statistical analyses.

## 3. Results

The results from the fours indices, LHR, MEI_SS_, MEI_LS_, PWV_mean_, and PEI, were first computed for the diabetic subjects with peripheral neuropathy within six years (i.e., Group 3) for comparison with the healthy elderly subjects (i.e., Group 1) and diabetic patients without peripheral neuropathy (i.e., Group 2). Subsequently, three new diabetic subgroups using different PEI values were identified for the goodness-of-fit test. Finally, the Cox regression analysis of risk factors for the incidence of DPN within six years after the PEI provided for diabetic patients was verified.

### 3.1. Comparison among LHR, MEI_SS_, MEI_LS_, PWV_mean_, and PEI for Age-Controlled Healthy and Diabetic Subjects with and without DPN 

After the entire follow-up process had been carried out and the DPN status was confirmed, the results from the comparison of the four previous computational parameters (i.e., LHR, MEI_SS_, MEI_LS_, and PWV_mean_) with the PEI for DPN identification assessment among the three groups of subjects are shown in Table 2. Although the value of PWV_mean_ was significantly higher in Group 2 compared with the Group 1 subjects (*p* < 0.017), there was no notable difference between Groups 2 and 3. On the other hand, the PEI showed highly significant differences among the three groups (*p* < 0.001) (Table 2).

### 3.2. Three Diabetic Subgroups Using Different Percussion Entropy Index (PEI) Values

The distribution of the PEI exhibited an approximately normal curve with a mild skew toward higher values. The values of the quartile ranges in the distribution were 0.27–0.54, 0.55–0.65, and 0.66–0.82 for the lower, middle two, and upper quartiles, respectively, for the prognostication of subjects with type 2 diabetes who are more prone to develop DPN. The diabetic patients in Group C showed remarkably higher HbA1c levels than those in the diabetic patients in Group B (*p* < 0.017). On the other hand, no significant differences were noted in the demographic and hemodynamic parameters, as well as the fasting blood glucose and serum lipid profile between any two groups (Table 3). In summary, a comparison of characteristics among subjects in the three categories revealed no significant differences in age, body mass index, waist circumference, systolic blood pressure, diastolic blood pressure, pulse pressure, high-density lipoprotein cholesterol, low-density lipoprotein cholesterol, and fasting plasma glucose.

### 3.3. Goodness-of-Fit Test and Cox Proportional Hazards Model for Relative Risks Analysis

#### 3.3.1. The Goodness-of-Fit Test

According to the chi-square goodness-of-fit test result in SPSS, the null hypothesis (i.e., no association between the PEI and DPN) was rejected with a chi-square value (i.e., the computed chi-square value, χ^2^ = 8.00) larger than the level of significance (χ^2^ = 5.99, α = 0.05). That is, diabetic patients with a smaller PEI value were associated with the future development of DPN within six years after the PEI was provided.

#### 3.3.2. Cox Proportional Hazards Model

A total of 27 type 2 diabetic patients developed DPN among 85 study patients (31.8%) within six years after the baseline examinations in this study. The progression to DPN in patients in the three categories within six years and corresponding relative risks for the incidence of DPN assessed by the Cox proportional hazards model are shown in Table 4. The Cox model revealed a graded association, with the diabetic subjects with a small PEI (i.e., Group C) at 2.90× greater risk of developing DPN on follow-up, relative to the diabetic subjects with a large PEI (i.e., Group A) after adjustment for entry age, waist circumference, BMI, systolic and diastolic blood pressure, total cholesterol, triglyceride, pulse pressure, high-density lipoprotein cholesterol, low-density lipoprotein cholesterol, glycosylated hemoglobin, and fasting blood sugar. In addition, the Cox model revealed a graded association, with the diabetic subjects with a moderate PEI (i.e., Group B) having almost equal risks for developing DPN on follow-up relative to the diabetic subjects with a large PEI (i.e., Group A).

### 3.4. Cox Regression Analysis

The regression analysis using the Cox proportional hazards regression analysis of risk factors for incidence of DPN is shown in Table 5. The PEI was also significantly associated with the risk of developing DPN when it was treated as a continuous variable. The relative risk of incidence of DPN within six years of follow-up in diabetic patients for the PEI was 4.77 (*p* = 0.015), whereas the relative risks of incidence of DPN for the value of fasting plasma glucose and glycosylated hemoglobin were 1.01 (*p* = 0.033) and 0.73 (*p* = 0.041), respectively. The term of interaction between HbA1c and FPG was not significant (*p* = 0.205).

Compared with fasting plasma glucose and glycosylated hemoglobin, smaller PEI values can provide valid information that may help identify type 2 diabetic patients at a greater relative risk of future DPN from baseline measurement (i.e., PEI provided) to the end of the follow-up period (i.e., within six years after the PEI was provided).

## 4. Discussion

Type 2 diabetes and its related complications are associated with the long-term damage and failure of various organ systems [31]. The overall impact of bad glucose control on vascular complications and major clinical outcomes in type 2 diabetes is still an open problem. While good glucose control has an undoubted benefit in the microvascular system of diabetic patients [6,7,8,32], good blood glucose control also improves microvascular disease and should be implemented early and maintained for the optimum length of time. A previous review study [31] highlighted the need for implementing programs for early detection, screening, and awareness to mitigate the burden of managing the complications. Therefore, diagnosis classifies a patient as having or not having a particular disease. In fact, diagnosis was recognized as the primary guideline for treatment and prognosis (i.e., what is going to happen in the future), and is still considered the key component of clinical practice [33]. However, it is not an easy job to control the appropriate glucose for diabetic patients; this is the reason DPN is still one of the most common chronic complications of diabetes. Recently, a study [23] that determined the exact PPI intervals was reported to provide a prognosis of peripheral neuropathy from diabetic patients. However, real-time computation was not able to obtain immediate index information of the test subjects. Furthermore, it did not provide valid information regarding the degree of risk of developing future DPN that may encourage type 2 diabetic patients to follow a good lifestyle.

This study addressed results from the indices LHR, MEI_SS_, MEI_LS_, PWV_mean_, and PEI, which were first computed for diabetic subjects with peripheral neuropathy six years after baseline measurement (i.e., Group 3) for comparison with diabetic patients without peripheral neuropathy (i.e., Group 2). Although the values of the vascular stiffness indices, including MEI_LS_ and PWV_mean_, were significantly different in Group 2 compared with the Group 1 subjects (*p* < 0.017), there were no notable differences between Groups 2 and 3 (*p* > 0.017). On the other hand, the PEI (i.e., BRS assessment index) showed highly significant differences among the three groups (*p* < 0.017) (Table 2). These results are consistent with the major outcomes in the previous study [23]. Significantly smaller values for the PEI were noted for Group 3 compared to the other two groups (e.g., Group 1 vs. Group 2 vs. Group 3: 0.73 ± 0.01 vs. 0.63 ± 0.01 vs. 0.59 ± 0.02), which is consistent with the same findings, where diabetic neuropathy was found to be a more significant crucial factor of spontaneous BRS assessment than carotid elasticity in type 2 diabetics in [34]. 

Therefore, most diabetic patients with a smaller PEI value were only concerned about the relative risks of the future development of DPN, and were not focused on achieving a smaller PEI value, because DPN, which has a lifetime prevalence of approximately 50%, is the most common diabetic complication. DPN is also the leading cause of disability due to diabetic foot ulceration and amputation, gait disturbance, and fall-related injury [3,35]. Neuropathy not only causes problems such as a decreased quality of life, poor sleep, and depression in diabetic patients, but the quality of life is also greatly affected [36,37,38]. Although PEI has recently been introduced to assess the complexity of BRS [21,22,23], the significance of smaller PEI values concerning the identification of subjects with type 2 diabetes who are more prone to develop diabetic neuropathy is unknown. That is, it would be difficult to predict how many and who will develop DPN in advance. Thus, a model of clinical practice focused on DPN prognosis and predicting the likelihood of future outcomes associated with PEI may be more useful for diabetic patients [33].

According to the results in Table 2, the present study adopted the PEI as the first measurement of all of the recruited diabetic patients to create the basis of quartiles in the diabetic population’s distribution of the PEI: the upper 25% (i.e., *n* = 22, Group A, 6 DPN included), the middle 50% (i.e., *n* = 42, Group B, 10 DPN included), and the lower 25% (i.e., *n* = 21, Group C, 11 DPN included). The diabetic patients in Group C showed remarkably higher HbA1c levels than those in the diabetic patients in Group B (*p* < 0.017) (Table 3). On the other hand, no significant differences were noted in the demographic and hemodynamic parameters, as well as the fasting blood glucose and serum lipid profile between any two groups (i.e., Group A vs. Group B and Group B vs. Group C) (Table 3). In the diabetic patients with smaller PEI values in Group 3, almost 50% had developed DPN within six years (Table 4). These results are consistent with statements in the study [31]. According to goodness-of-fit test result in the study, the null hypothesis (i.e., no association between smaller PEI values and developing DPN within six years after the PEI was provided) was rejected, with the chi-square value being larger than the level of significance. An association exists between diabetic patients with smaller PEI values and the development of DPN within six years after baseline measurement.

A total of 27 type 2 diabetic patients developed DPN among 85 study patients (31.8%) in the six years after baseline examinations. This finding is consistent with similar results reported in [37,38]. The progression to DPN of patients in the three categories within six years and the corresponding relative risks for the incidence of DPN assessed by the Cox proportional hazard survival model are shown in Table 4. The Cox model revealed a graded association, with the diabetic subjects with a small PEI (i.e., Group C) at 2.90-times greater risk of developing DPN on follow-up relative to the diabetic subjects with a large PEI (i.e., Group A) after adjustment for entry age, waist circumference, BMI, systolic blood pressure and diastolic blood pressure, total cholesterol, triglyceride, pulse pressure, high-density lipoprotein cholesterol, low-density lipoprotein cholesterol, glycosylated hemoglobin, and fasting plasma glucose. Type 2 diabetic patients with smaller PEI values may have a larger relative risk of developing DPN within six years. In addition, the relative risk of incidence of DPN within six years of follow-up in diabetic patients for the PEI was 4.77 (*p* = 0.015) in Table 5. This study was designed to use synchronized ECG and PPG signals (i.e., PEI) in predicting the development of peripheral neuropathy from type 2 diabetes. The PEI has recently been introduced not only to assess the complexity of BRS but also to show the significance of smaller PEI values concerning the identification of subjects with type 2 diabetes who are more prone to DPN.

The current study has its limitations. Firstly, the number of subjects recruited was relatively small. In addition, it may be difference between DPN attack confirmation time and checkout time in our study for non-fixed follow-up to each diabetic patient. Therefore, Kaplan–Meier survival analysis was not adopted in this study. Nevertheless, highly significant associations between PEIs and relative risks of developing DPN were still significant. Secondly, this study focused on the Cox proportional hazard survival model for diabetic patients, and the optimal BRS delay between the amplitude and RRI series would be set at one to five heartbeat cycles for all test subjects with the same setting. Thirdly, the impact of periodontal therapy on diabetes control was not investigated because of the limited number of diabetic patients. Subsequently, the period of baseline measurement was more than two years, because of the limited number of subjects in each group. Finally, as an observational study, the values of PEI and the proposed parameters could be used to identify the risk factors for a prediction task by using simple machine learning algorithms (such as SVM, LDA, or even deep learning) in the future.

## 5. Conclusions

This study represents the first attempt to investigate the clinical prognostic feasibility of applying the PEI, and demonstrates enhanced sensitivity in differentiating between diabetic subjects without DPN and diabetic subjects with DPN within six years after baseline measurement, compared to single-scale indices (i.e., LHR and PWV_mean_) and multiple temporal-scale indices (i.e., MEI_SS_ and MEI_LS_). Our findings suggest that diabetic patients with smaller PEI values are more prone to developing DPN, which is of potential importance for application in the area of the point-of-care diagnostic devices.

## Figures and Tables

**Table 1:** Anthropometric, hemodynamic, and serum biochemical parameters of the testing subjects.

Parameters	Group 1 (*n* = 37) Female/Man (19/18)	Group 2 (*n* = 58) Female/Man (24/34)		Group 3 (*n* = 27) Female/Man (13/14)	
**Age, years**	59.20 ± 1.67	61.80 ± 1.45	(*p* = 0.66)	62.81 ± 1.71	(*p* = 0.22)
**Body height, cm**	161.10 ± 1.19	160.37 ± 1.04	(*p* = 0.65)	164.15 ± 1.78	(*p* = 0.06)
**Body weight, kg**	60.95 ± 1.66	68.86 ± 1.45 **	(*p* = 0.00)	72.48 ± 1.46	(*p* = 0.13)
**WC, cm**	82.19 ± 1.77	93.32 ± 1.30 **	(*p* = 0.00)	96.50 ± 1.45	(*p* = 0.14)
**BMI, kg/m** ^**2**^	23.47 ± 0.59	26.82 ± 0.57 **	(*p* = 0.00)	27.08 ± 0.75	(*p* = 0.79)
**SBP, mmHg**	123.17 ± 3.21	125.96 ± 2.28	(*p* = 0.47)	127.85 ± 6.27	(*p* = 0.73)
**DBP, mmHg**	74.69 ± 1.55	74.91 ± 1.29	(*p* = 0.91)	73.23 ± 3.45	(*p* = 0.58)
**PP, mmHg**	48.49 ± 2.41	50.13 ± 2.08	(*p* = 0.61)	54.62 ± 3.73	(*p* = 0.26)
**HDL, mg/dL**	52.01 ± 3.59	44.65 ± 2.62	(*p* = 0.09)	42.79 ± 3.77	(*p* = 0.69)
**LDL, mg/dL**	114.65 ± 5.08	120.77 ± 6.48	(*p* = 0.49)	106.58 ± 4.90	(*p* = 0.15)
**Cholesterol, mg/dL**	192.16 ± 8.33	177.02 ± 7.65	(*p* = 0.19)	183.60 ± 7.01	(*p* = 0.58)
**Triglyceride, mg/dL**	92.45 ± 6.08	144.61 ± 11.44 **	(*p* = 0.00)	161.04 ± 13.47	(*p* = 0.39)
**HbA1c, %**	5.90 ± 0.06	8.12 ± 0.23 **	(*p* = 0.00)	8.36 ± 0.30	(*p* = 0.54)
**FPG, mg/dL**	99.80 ± 4.42	149.46 ± 6.59 **	(*p* = 0.00)	161.44 ± 11.26	(*p* = 0.33)

Values are expressed as mean ± SD. Group 1, healthy elderly subjects; Group 2, diabetic subjects; Group 3, diabetic subjects with peripheral neuropathy 6 years after baseline measurement. The total number of test subjects was 122. WC, waist circumference; BMI, body mass index; SBP, systolic blood pressure; DBP, diastolic blood pressure; PP, pulse pressure; HDL, high-density lipoprotein cholesterol; LDL, low-density lipoprotein cholesterol; HbA1c, glycosylated hemoglobin; FPG, fasting plasma glucose. ** *p* < 0.001 Group 1 vs. Group 2. *p* values of the parameter larger than 0.017 are regarded as not statistically significant between two groups. The total number of subjects is 122 in this table.

**Table 2:** Of computational parameters for autonomic function assessment in three groups of testing subjects.

Parameters	Group 1 (*n* = 37)	Group 2 (*n* = 58)		Group 3 (*n* = 27)	
**LHR**	1.56 ± 0.17	2.00 ± 0.26	(*p* = 0.23)	2.34 ± 0.44	(*p* = 0.49)
**MEI_ss_**	0.62 ± 0.08	0.57 ± 0.02 *	(*p* = 0.01)	0.55 ± 0.16	(*p* = 0.72)
**MEI_Ls_**	1.56 ± 0.06	1.48 ± 0.04	(*p* = 0.31)	1.37 ± 0.06	(*p* = 0.69)
**PWV_mean_**	4.65 ± 0.06	4.93 ± 0.06 *	(*p* = 0.01)	4.80 ± 0.07	(*p* = 0.22)
**PEI**	0.73 ± 0.01	0.63 ± 0.01 **	(*p* = 0.00)	0.59 ± 0.02 ^†^	(*p* = 0.01)

Values are expressed as mean ± SD. Group 1, healthy elderly subjects; Group 2, diabetic subjects; Group 3, diabetic subjects with peripheral neuropathy within six years after baseline measurement. LHR, low- to high-frequency power ratio; MEI_SS_, small-scale multiscale entropy index, MEI_LS_, large-scale multiscale entropy index, PWV_mean_, ECG-PWV-based pulse wave velocity; PEI, percussion entropy index. * *p* < 0.017 (*p* corrected), Group 1 vs. Group 2; ** *p* < 0.001, Group 1 vs. Group 2; ^†^ *p* < 0.017, Group 2 vs. Group 3. The total number of subjects in this table is 122.

**Table 3:** Demographic, anthropometric, hemodynamic, and serum biochemical parameters of the testing diabetic patients in Groups 2 and 3.

Parameters	Group A (*n* = 22) Female/Man (8/14)	Group B (*n* = 42) Female/Man (20/22)		Group C (*n* = 21) Female/Man (9/12)	
Age, year	65.62 ± 1.62	64.95 ± 2.22	(*p* = 0.36)	63.00 ± 2.30	(*p* = 0.81)
Body height, cm	160.99 ± 1.32	162.90 ± 1.86	(*p* = 0.82)	161.53 ± 1.87	(*p* = 0.41)
Body weight, kg	68.98 ± 1.58	70.53 ± 9.37	(*p* = 0.32)	71.79 ± 2.30	(*p* = 0.57)
WC, cm	93.56 ± 1.51	94.10 ± 1.78	(*p* = 0.29)	96.32 ± 2.00	(*p* = 0.83)
BMI, kg/m^2^	26.67 ± 0.61	26.61 ± 0.78	(*p* = 0.38)	27.73 ± 1.12	(*p* = 0.95)
SBP, mmHg	125.38 ± 4.13	130.63 ± 4.83	(*p* = 0.97)	125.16 ± 2.84	(*p* = 0.45)
DBP, mmHg	72.71 ± 2.26	75.58 ± 2.66	(*p* = 0.26)	76.79 ± 1.79	(*p* = 0.46)
PP, mmHg	52.67 ± 2.72	52.30 ± 4.53	(*p* = 0.32)	48.37 ± 2.10	(*p* = 0.94)
HDL, mg/dL	45.11 ± 3.19	46.67 ± 5.04	(*p* = 0.22)	38.88 ± 2.29	(*p* = 0.79)
LDL, mg/dL	110.30 ± 6.04	122.11 ± 11.42	(*p* = 0.27)	122.12 ± 8.64	(*p* = 0.32)
Cholesterol, mg/dL	171.53 ± 7.29	186.47 ± 10.13	(*p* = 0.22)	188.83 ± 13.38	(*p* = 0.25)
Triglyceride, mg/dL	148.97 ± 10.80	139.89 ± 14.50	(*p* = 0.57)	162.28 ± 24.77	(*p* = 0.63)
HbA1c, %	7.83 ± 0.24	8.40 ± 0.41	(*p* = 0.03)	8.78 ± 0.32 ^†^	(*p* = 0.01)
FPG, mg/dL	148.84 ± 8.27	158.56 ± 13.71	(*p* = 0.47)	158.82 ± 8.78	(*p* = 0.53)

Group A, diabetic subjects with large PEI (the upper 25%); Group B, diabetic subjects with moderate PEI (the middle 50%); Group C, diabetic subjects with small PEI (the lower 25%). Values are expressed as mean ± SD. WC, waist circumference; BMI, body mass index; SBP, systolic blood pressure; DBP, diastolic blood pressure; PP, pulse pressure; HDL, high-density lipoprotein cholesterol; LDL, low-density lipoprotein cholesterol; HbA1c, glycosylated hemoglobin; FPG, fasting plasma glucose. ^†^ *p* < 0.017 (*p* corrected), Group B vs. Group C. The total number of patients in this table is 85.

**Table 4:** Progression to DPN within six years of follow-up and relative risks as a function of three categories of PEI.

Categories of PEI Values	Subjects at Risk (*n*)	Events of DPN (*n*)	Relative Risk (95% CI)
**Group A**	22	6	1.00 (reference)
**Group B**	42	10	0.95 (0.63–2.07)
**Group C**	21	11	2.90 (1.58–6.87)
Total	85	27	—

Group A, diabetic subjects with a large PEI (the upper 25%); Group B, diabetic subjects with a moderate PEI (the middle 50%); Group C, diabetic subjects with a small PEI (the lower 25%). Relative risk estimated from a Cox proportional hazards survival model with adjustment for entry age, body mass index, resting systolic and diastolic blood pressure, total cholesterol, triglyceride, pulse pressure, high-density lipoprotein cholesterol, low-density lipoprotein cholesterol, and glycosylated hemoglobin. DPN, diabetic peripheral neuropathy; CI, confidence interval. The Cox proportional hazard survival model in SPSS was adopted. Events of DPN, number of future developing peripheral neuropathy in type 2 diabetic subjects within six years.

**Table 5:** A proportional hazards analysis of risk factors for incidence of DPN within six years of follow-up in diabetic patients.

Risk Factors	Relative Risk	95% CI	*p* Values
**HbA1c, %**	0.73	0.52–1.05	0.041
**FPG, mg/dL**	1.01	1.00–1.02	0.033
**HbA1c× FPG**	1.00	1.00–1.01	0.205
**PEI**	4.77	1.87–6.31	0.015

HbA1c, glycosylated hemoglobin; FPG, fasting plasma glucose; PEI, percussion entropy index; DPN, diabetic peripheral neuropathy; CI, confidence interval. The variables of the models are entry age, body mass index, resting systolic and diastolic blood pressure, total cholesterol, triglyceride, pulse pressure, high-density lipoprotein cholesterol, low-density lipoprotein cholesterol, and glycosylated hemoglobin from baseline to the end of follow-up. Cox proportional hazards regression analysis in SPSS was adopted. A *p*-value < 0.05 was noted as statistically significant.

## Abbreviations

BMI	Body Mass Index
BRS	Baroreflex Sensitivity
CI	Confidence Interval
DBP	Diastolic Blood Pressure
DM	Diabetes Mellitus
DPN	Diabetic Peripheral Neuropathy
DVP	Digital Volume Pulse
ECG	Electrocardiography
FPG	Fasting Plasma Glucose
HbA1c	Glycosylated hemoglobin
HDL	High-Density Lipoprotein cholesterol
HFP	High Frequency Power
HRV	Heart Rate Variability
LDL	Low-Density Lipoprotein cholesterol
LFP	Low-Frequency Power
LHR	Low-/High-frequency power Ratio (LFP/HFP, LHR) using the RRI dataset
MEI	Multiscale Entropy Index using the RRI dataset only
PEI	Percussion Entropy Index using synchronized {RRI} and {Amp} signals
PN	Peripheral Neuropathy
PP	Pulse Pressure
PPG	Photoplethysmography
PPI	Peak-to-Peak Interval
PWV	Pulse Wave Velocity
RR	Relative Risks
RRI	R-R Interval of ECG
SBP	Systolic Blood Pressure
SD	Standard Deviation
SPSS	Statistical Package for the Social Sciences
TG	Triglyceride
WC	Waist Circumference
